# The Gut Microbiota of the Insect Infraorder Pentatomomorpha (Hemiptera: Heteroptera) for the Light of Ecology and Evolution

**DOI:** 10.3390/microorganisms9020464

**Published:** 2021-02-23

**Authors:** Hongwei Shan, Wei Wu, Zongtao Sun, Jianping Chen, Hongjie Li

**Affiliations:** State Key Laboratory for Managing Biotic and Chemical Threats to the Quality and Safety of Agro-products, Key Laboratory of Biotechnology in Plant Protection of Ministry of Agriculture and Zhejiang Province, Institute of Plant Virology, Ningbo University, Ningbo 315211, China; shanhongwei@nbu.edu.cn (H.S.); wuwei_19861115@163.com (W.W.); sunzongtao@nbu.edu.cn (Z.S.); jianpingchen@nbu.edu.cn (J.C.)

**Keywords:** stinkbugs, symbiotic organ, gut symbionts, insect–microbe interactions

## Abstract

The stinkbugs of the infraorder Pentatomomorpha are a group of important plant sap-feeding insects, which host diverse microorganisms. Some are located in their complex morphological midgut compartments, while some within the specialized bacteriomes of insect hosts. This perpetuation of symbioses through host generations is reinforced via the diverse routes of vertical transmission or environmental acquisition of the symbionts. These symbiotic partners, reside either through the extracellular associations in midgut or intracellular associations in specialized cells, not only have contributed nutritional benefits to the insect hosts but also shaped their ecological and evolutionary basis. The stinkbugs and gut microbe symbioses present a valuable model that provides insights into symbiotic interactions between agricultural insects and microorganisms and may become potential agents for insect pest management.

## 1. Introduction

Hemiptera comprises stinkbugs, aphids, whiteflies, psyllids, planthoppers, leafhoppers, and cicadas, etc., are not only the most important agricultural pests, but also the common vectors that spread plant pathogenic virus, bacteria, phytoplasma and fungi, which lead to severe economic impacts in major crop plants worldwide [1,2,3,4]. Major hemipteran insects feed on phloem sap or xylem sap that are nutritionally poor or unbalanced diets for the insect hosts live throughout the life cycle, and consequently, symbiotic associations with beneficial microorganisms to provide a supplementary source of nutrients including essential amino acids and/or vitamins [5,6,7]. As the exquisite evolutionary adaptations, hemipteran insects have evolved specialized cells, tissues, and/or organs to accommodate and maintain symbiotic microorganisms, such as specialized intestines and even cells called bacteriocytes [8,9]. Most Sternorrhyncha and Auchenorrhyncha includes aphids, whiteflies, and leafhoppers, are associated with primary/obligate symbiotic relationship with bacteria that are presented in the bacteriocytes within the insect body [10]. In contrast, many stinkbugs (Hemiptera: Heteroptera) are lack of bacteriocytes, instead their posterior region of midguts are morphologically differentiated into alternative habitats for housing symbiotic microbiota [11,12]. In addition, various facultative symbionts have been found to provide the insect hosts with parasitoids resistance, heat tolerance or insecticide resistance [13,14,15,16], which lead hemipteran insects a tremendously ecological success. These insects maintain these specific beneficial partners between generations either via vertically acquire from parents or horizontally acquire from the environment with diverse routes [17]. Herein, we extensively review the literatures on the mutualistic associations between stinkbugs and their microbial symbionts, therefore provide deeper insights into the ecology and evolution of the gut microbiota in these insects. Specifically, we include (*i*) the evolutionary trajectory for hosting microbial symbionts across hemipteran insects; (*ii*) the diverse strategies of the microbial acquisition; and (*iii*) its ecological implications and potentials as a studying model for insect–gut microbiota interactions.

## 2. Evolutionary Trajectory for Hosting Microbial Symbionts in Hemiptera

**Figure 1:**
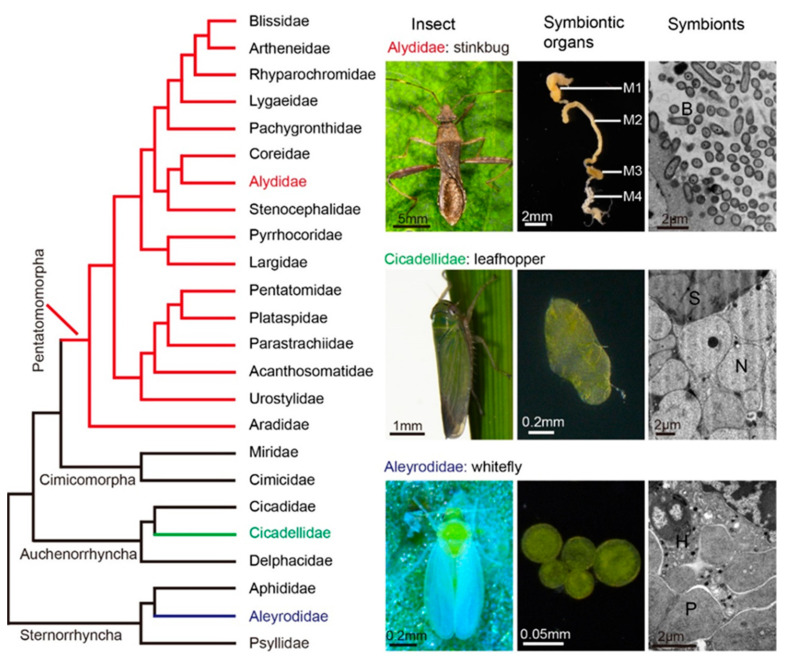
Phylogeny of stinkbugs in Hemiptera. Left panel: A simplified phylogeny of the pentatomomorphan stinkbugs in Hemiptera. The red colored lines indicate the families of stinkbugs. The tree is based on the references [6,18] with slight modification. Right panel: The insect hosts, their symbiotic organs and bacterial symbionts in three representative families of the suborders Heteroptera (the infraorder Pentatomomorpha), Auchenorrhyncha, and Sternorrhyncha. Top images (from left to right): an alydid stinkbug *Riptortus pedestris*, the four sections of the midgut, and the transmission electron micrographs (TEM) of *Burkholderia* symbiont in midgut crypts. M1, midgut first section; M2, midgut second section; M3, midgut third section; M4, midgut fourth section with crypts; B: *Burkholderia*. Middle images: a planthopper *Laodelphax striatellus,* bacteriome and the micrograph of their dual obligate symbionts *Nasuia* and *Sulcia* in bacteriome. N: *Nasuia*; S: *Sulcia*. Bottom images: A whitefly *Bemisia tabaci*, bacteriocytes, and the micrograph of obligate symbiont *Portiera* and facultative symbiont *Hamiltonella* in bacteriocyte. P: *Portiera*; H: *Hamiltonella*.

Mutualistic associations between hemipteran insects and microorganisms are quite omnipresent, particularly in the suborders Sternorrhyncha, Auchenorrhyncha and Heteroptera (with infraorders Cimicomorpha and Pentatomomorpha) (Figure 1) [6,18]. Moreover, these insects have evolved different symbiotic tissues for housing the symbionts over evolutionary time.

### 2.1. The intracellular Symbiotic Association of Sternorrhyncha

Obligate intracellular symbioses are dominant in the Sternorrhyncha (aphids, whiteflies, psyllids, and mealybug, etc.), and the symbionts are mostly restricted in the host specialized cells so-called bacteriocytes (Figure 1 and Table 1) [19,20,21,22,23,24,25,26,27,28,29]. In the well-studied aphids–*Buchnera* symbiosis, the obligate endosymbionts are embedded in the bacteriocytes located within the body cavities of insect hosts [30]. The symbionts are permanently residing in the host bacteriocytes throughout all insect developmental stages, except for the host parthenogenetic reproduction, in which symbionts will leave the bacteriocytes and transmit into the embryo [31]. The intracellular lifestyles drive the genomes of obligate symbionts towards AT-biased nucleotide composition and with significant reduction [32,33]. While the symbiont also retains the genes for the synthesis of all essential amino acids that are almost completely depleted in plant phloem diet of the host [21]. Most aphids contain the obligate *Buchnera*, but it is absent in some Cerataphidini aphids and replaced by a yeast-like symbiont located in the abdominal hemocoel within the body cavity [34]. In addition, many facultative symbionts are co-inhabited with *Buchnera* in aphids, such as *Hamiltonella defensa*, *Serratia symbiotica,* and *Regiella insecticola*, are grouped into clusters within secondary bacteriocytes located between the primary bacteriocytes [14]. These facultative symbionts do not appear to perform essential nutritional functions but play important roles in ecological adaptation for host [14,35,36].

The family Aleyrodidae whiteflies, such as *Bemisia tabaci* and *Aleurodicus dugesii*, are major agricultural pests causing plant damage and transmitting plant viruses. The whiteflies have dozens of relatively small, roundish and orange bacteriocytes that contain a pleiomorphic obligate bacterium *Portiera aleyrodidarum* (Figure 1) [37]. They have a unique mode for transmitting bacteriocyte symbionts to progeny, in which the intact bacteriocytes migrate to the ovaries and enter the eggs [38,39]. Moreover, the insects contain several facultative symbionts with scattered distributions. Some symbionts like *Hamiltonella defensa*, *Wolbachia* sp., or *Arsenophonus* sp. share the same symbiotic cell with *Portiera* and co-located in the cytoplasm of bacteriocyte (Figure 1) [40]. The obligate *Portiera* provides its host with essential amino acids and carotenoids and while one or several facultative symbionts involve in synthesizing B vitamins and other cofactors [41,42]. Moreover, another facultative symbiont *Rickettsia* resides in remarkable habitats within whitefly hosts, in which the bacteria occupy most of the insect body cavities that are widespread in the bacteriocytes, midgut, fat body, hemocytes, and hemolymph [43,44,45]. However, these sap-feeding insects include aphids and whiteflies are found as almost completely absent of gut microbiota [5,46], or as for whiteflies, only associated with some intracellular *Rickettsia* symbionts that restrictedly inhabit within the midgut epithelial cells [44].

### 2.2. The Intracellular Symbiotic Association of Auchenorrhyncha

Most groups of the Auchenorrhyncha including leafhoppers (the family Cicadellidae), cicadas (the family Cicadidae) and planthoppers (the family Delphacidae) are phloem or xylem feeders, and evolve a pair of symbiotic tissues constitute several specialized cells form a larger structure, often referred as bacteriomes. Unlike the exclusive obligate symbiosis in most Sternorrhyncha, the leafhoppers and cicadas often have dual obligate the symbionts, and usually harbor the obligate symbiont *Sulcia muelleri* with one other kind of the co-obligate symbiont, e.g., *Baumannia cicadellinicola* or *Nasuia deltocephalinicola* in the bacteriomes (Figure 1) [25,47]. In general, the *Sulcia* has the capacity to synthesize eight of the 10 essential amino acids and another co-obligate symbiont enable to produce the remaining two essential amino acids and other vitamins [25,48]. While symbiont replacements or losses have occurred in some leafhoppers. In the subfamily Ledrinae, *Ledra auditura* Walker only harbors *Sulcia* symbiont and the co-obligate symbiont is replaced by yeast-like fungal symbionts within insect fat bodies [49]. Furthermore, some leafhoppers species even lack both obligate symbionts and instead harbor *Cardinium* symbionts and/or the yeast-like symbiont in their fat bodies to fulfill the nutritional roles for hosts [49,50]. Intriguingly, in the planthoppers (family Delphacidae), those insects are not associated with specifically obligate bacterial symbionts and the obligate association is replaced by the yeast-like symbionts involved in nitrogen recycling of hosts to fulfill the nutritional roles [28]. Meanwhile, a facultative symbiont *Wolbachia*, a famous reproductive parasite in arthropods, is frequently inhabited in these insects involved in the metabolism of B vitamins to enhance the fecundity of female host insects [51].

**Table 1:** The nutrient related symbionts and their localizations in symbiotic organs of representative the hemipteran insects.

Insects		Symbionts	Obligate	Localization	Transmission Routes	References
Suborder/Infraorder/Superfamily	Family					
Sternorrhyncha	Psyllidae	*Carsonella ruddii*	+	Bacteriocyte	Ovarial passage	[19]
	Aleyrodidae	*Portiera aleyrodidarum*	+	Bacteriocyte	Ovarial passage	[20]
	Aphididae	*Buchnera aphidicola*	+	Bacteriocyte	Ovarial passage	[21]
	Pseudococcidae	*Tremblaya princeps*	+	Bacteriocyte	Ovarial passage	[22]
Cicadomorpha	Cicadellidae	*Sulcia muelleri* and *Baumannia cicadenillicola* or *Nasuia koganicola*	+	Bacteriocyte	Ovarial passage	[23,24]
	Cicadidae	*S. muelleri* and *Hodgkinia cicadicola*	+	Bacteriocyte	Ovarial passage	[25]
	Membracidae	*S. muelleri* and *Nasuia koganicola*	+	Bacteriocyte	Ovarial passage	[26]
	Cercopidae	*S. muelleri* and *Zinderia insecticola*	+	Bacteriocyte	Ovarial passage	[27]
Fulgoromorpha	Delphacidae	Yeast-like symbionts	+	Fat body	Ovarial passage	[28]
	Cixiidae	*S. muelleri* and *Vidania fulgoroideae*	+	Bacteriocyte	Ovarial passage	[29]
Cimicomorpha	Cimicidae	*Wolbachia*	+	Bacteriocyte	Ovarial passage	[52,53]
	Miridae	*Rickettsia* and *Wolbachia*	−	Bacteriocyte &Midgut	Ovarial passage	[54,55]
Pentatomomorpha						
Pentatomoidea	Pentatomidae	*Pantoea* spp.	+	Midgut (M4)	Egg surface	[56]
	Plataspidae	*Ishikawaella capsulatus*	+	Midgut (M4)	Capsule	[57]
	Parastrachiidae	*Benitsuchiphilus tojoi*	+	Midgut (M4)	Egg surface	[58,59]
	Acanthosomatidae	*Rosenkranzia clausaccus*	+	Midgut (M4)	Egg surface	[60]
	Urostylidae	*Tachikawaea gelatinosa*	+	Midgut (M4)	Jelly	[61]
	Scutelleridae	*Pantoea* spp.	+	Midgut (M4)	Egg surface	[62]
Lygaeoidea	Blissidae	*Ischnodemia utricula/Burkholderia* spp.	+/−	Bacteriocyte/Midgut (M4)	Ovarial passage/Environment	[63,64]
	Artheneidae	*Rohrkolberia cinguli*	+	Midgut (M1)	Ovarial passage	[65]
	Rhyparochromidae	*Burkholderia* spp.	−	Midgut (M4)	Environment	[66]
	Lygaeidae	*Kleidoceria schneideri/* *Schneideria nysicola*, etc.	+	Bacteriocyte	Ovarial passage	[63,67,68]
Coreoidea	Coreidae	*Burkholderia* spp.	−	Midgut (M4)	Environment	[66]
	Alydidae	*Burkholderia* spp.	−	Midgut (M4)	Environment	[65]
	Stenocephalidae	*Burkholderia* spp.	−	Midgut (M4)	Environment	[69]
Pyrrhocoroidea	Pyrrhocoridae	*Coriobacterium glomerans,* *Gordonibacter* sp., etc.	−	Midgut (M3)	Egg surface	[7,70,71]
	Largidae	*Burkholderia* spp.	−	Midgut (M4)	Environment	[72,73]

Note: the nutritional roles of *Burkholderia* and some bacteriocyte symbionts of super family Lygaeoidea are speculated by their genomes and related to host beneficial fitness to insect hosts. “+” means obligate symbiont; “−” means facultative symbiont. The four superfamilies of the stink bugs (Pentatomomorpha) are listed below.

### 2.3. The Intracellular and Extracellular Symbiotic Associations of Heteroptera

Among the Heteroptera, two infraorders Cimicomorpha and Pentatomomorpha including blood and plant sucking bugs that are universally associated with diverse symbionts. In the Cimicomorpha, the bedbug *Cimex lectularius* (family Cimicidae) harbors *Wolbachia* in the bacteriocytes to supply the B vitamins that are devoid in their blood meal but essential for host’s growth and reproduction [52,53]. The reduviid bugs *Rhodnius prolixus* and *Triatoma infestans* (family Reduviidae) lack bacteriomes and harbor symbiotic Actinobacteria *Rhodococcus rhodnii* and *Nocardia* sp. in the anterior midgut regions, e.g., cardia and stomach, are required for the development and survival of the insect hosts [74,75]. Intriguingly, some predatory Mirid bugs (family Miridae) are also associated with *Wolbachia* and *Rickettsia* in their bacteriocytes and epithelial cells that may provide nutritional benefits to their hosts (Table 1) [54,55].

**Figure 2:**
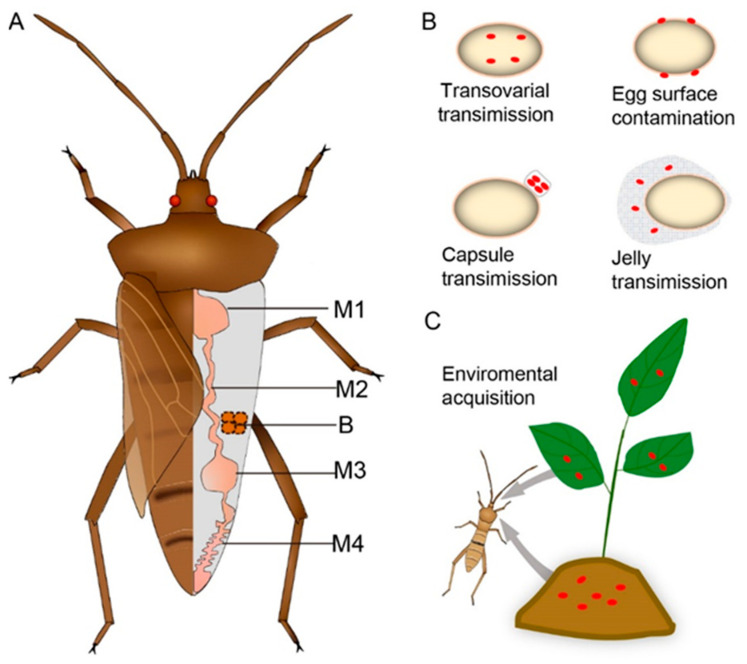
The main transmission routes of symbionts in stinkbugs. (**A**) schematic stinkbug with symbiotic tissues in an adult insect. M1, midgut first section; M2, midgut second section; M3, midgut third section; M4, midgut fourth section with crypts; B, bacteriomes, dotted line means that bacteriomes are absent in some species. (**B**) four distinct routes for the vertical transmission of symbionts during the egg stage; (**C**) the bacterial acquisition from soil or plant material during the nymph stage.

Various stinkbugs within the members of infraorder Pentatomomorpha are known as a notorious agricultural pest that damages diverse crop plants worldwide. Unlike the Sternorrhyncha and Auchenorrhyncha, the intestinal bacterial microbiota in the Pentatomomorpha is rather diverse, and the majority of these group insects lack bacteriocytes, only with exception of some Lygaeoidea lineages (Figure 2A). The midgut of stinkbugs has evolved more complex gut morphologies and differentiated into four distinct sections (Figure 1 and Table 1) [7,56,57,58,59,60,61,62,63,64,65,66,67,68,69,70,71,72,73]. These insects also possess specialized tissue in the posterior region of the midgut (e.g., the midgut fourth section refers to M4, Figure 1) that is morphologically modified into substantial sac-like structures called crypts, in which harbor bacterial symbionts [11,12], and usually, only single bacterial lineage dominates in the M4 crypts for many stinkbug hosts. In Pentatomoidea superfamily, insects do not harbor bacteriocyte symbionts but instead have many different types of obligate gut symbionts (Table 1). Specifically, the *Ishikawaella capsulata* is associated with plataspid stinkbugs and locate in the M4 of midgut crypts. According to the phylogenetic analysis, this symbiont is the sister group of the aphid obligate symbiont *Buchnera*, and exhibits AT-biased nucleotide composition and with reduced genome size. Furthermore, these specific gut symbionts are also found to be correlated with the growth, mortality, and sterility of the insect hosts [57]. Collectively, these lines of evidence indicate that the extracellular symbionts are likely to be the obligate partners of the stinkbugs. A variety of obligate gut symbionts are reported in other families of the superfamily Pentatomoidea stinkbugs, such as *Tachikawaea gelatinosa* in urostylid insects, *Pantoea* spp. in pentatomid insects, and *Rosenkranzia clausaccus* in the acanthosomatid insects (Table 1), which are all specifically located in the midgut crypts of each host. In the stinkbugs of the family Scutelleridae, the symbionts were close to the pentatomid gut symbionts of the genus *Pantoea* is extracellular in the midgut cavity of M4, whereas a facultative symbiont *Sodalis* sp. is also regularly found in some species associated with the host gonads [62,76,77]. Among the stinkbugs, *Burkholderia* spp. is the most widespread symbiont and extensively found association in M4 crypts of various insect groups in three stinkbugs superfamilies (Lygaeoidea, Coreoidea, and Pyrrhocoroidea) [66,72,78]. Although the *Burkholderia* symbionts are not strictly obligate with these bug species, these bacteria have been reported to benefit the development, survival, as well as body size and weight of insect hosts, indicating their roles as beneficial mutualists [79].

In addition to crypts of M4 region, the other regions of the midgut are also the habitats for the symbiotic microorganisms in some firebugs and cotton stainers (i.e., family Pyrrhocoridae). These stinkbugs are characterized by the absence of crypts in the M4 region, but instead the substantial gut microbiota is concentrated in the anterior part of midgut section M3, with bacteria cells occurring both attached to the epithelium and free-floating in the host gut lumen [70]. The microbiota mainly consists of Actinobacteria (e.g., *Coriobacterium glomerans*), Firmicutes (e.g., *Clostridium* sp.), and Proteobacteria (e.g., *Klebsiella* sp.). These symbionts are not obligate partners but also play essential roles in host fitness [7,71]. Moreover, the analysis of gut microbiota community structures reveals diverse transient bacterial lineages, such as *Brevundimonas*, *Rhizobium*, *Pseudomonas,* and *Caulobacter* species, residing in the M1, M2, and M4 regions of the pyrrhocorid stinkbug midgut [80].

In contrast, the stinkbugs of superfamily Lygaeoidea are atypical groups that have a promiscuous distribution with intracellular or extracellular symbionts. In the family Artheneidae, a stinkbug *Chilacis typhae* has the enlarged midgut epithelial cells at the end of the M1 section, and the endosymbiont *Rohrkolberia cinguli* locates in the cytoplasm of the epithelial cells [65]. In addition, the stinkbugs of the family Lygaeidae are harboring endosymbiotic bacteria embedded by specialized bacteriomes. It has been proposed that these species of stinkbugs evolved bacteriome-associated endosymbionts independently through the evolutionary time, such as the symbiont *Kleidoceria schneideri* in the bacteriome of a birch catkin bug *Kleidocerys resedae*, the symbiont *Rohrkolberia belonochilicola* in the bacteriome of *Belonochilus numenius*, and the symbiont *Schneideria nysicola* in the bacteriome of *Orsillus depressus* [63,67]. Regardless of the bacteriome-associated endosymbiont in these group stinkbugs, most lygaeoid and rhyparochromid stinkbugs are consistently associated with extracellular *Burkholderia* symbionts in the M4 crypts [66]. More interestingly, the stinkbugs of the family Blissidae either harbor the bacteriome-associated symbionts or gut symbionts in M4 crypts. For example, *Ischnodemus sabuleti* stinkbug is obligately associated with the bacteriocyte-symbionts *Ischnodemia utricula*, whereas *Blissus insularis* stinkbug has the *Burkholderia* in their M4 section [63,64].

## 3. The Diverse Strategies of Gut Microbiota Acquisitions in the Stinkbugs

Stinkbugs have been reported to acquire the gut bacterial microbiota through at least five measures throughout their developmental stages. In general, the gut microbiota may transfer to their offspring either via transovarial transmission during oogenesis, or via the exterior of the egg through smearing egg surface, symbiont capsule, and egg jelly during oviposition, or via horizontal acquisitions from the environment during the nymph stage (Figure 2).

### 3.1. The Vertical Transmission of the Gut Microbiota in the Stinkbugs

Transovarial transmission is likely crucial for establishing intracellular microorganisms, as the symbionts use ovarian passage to enter the egg to accomplish their transmission during pre-oviposition (Figure 2B). Although most symbiotic microorganisms are extracellularly associated of the intestine in stinkbugs, the symbiont *Rohrkolberia* is exceptionally endobiotic in the cytoplasm of midgut epithelial cells locating in the M1 of lygaeid stinkbug *C. typhae*. The endosymbionts can be transferred to the follicle cells of the germarium and then enter the oocyte cytoplasm, providing strong evidence for transovarial transmission within the egg [65]. The ovarian route of the symbiont transfer occurs in an enclosed environment, within the insect host bodies, facilitating reliable transmission and enable the bacteria to avoid various environmental stresses.

The extracellular symbionts are usually transferred via the exterior of the eggs and then the nymphs acquire the maternal symbionts after hatch. A variety of stinkbugs have been reported that smear their gut symbionts from the anus on the egg surface to accomplish the transfer, particularly among members of superfamily Pentatomoidea and Pyrrhocoroidea (Figure 2B, Table 1). For example, firebugs (the family Pyrrhocoridae) deposit their gut (M3) symbionts *Coriobacterium* and *Gordonibacter* sp. from the main tract of midgut to the surface of the eggs during oviposition, consequently followed by newly hatched nymphs probe the egg surface with their proboscis to acquire these symbionts, facilitating the mutual relationships with symbionts between generations [70]. Notably, these bacteria are often detected in the insect feces, indicating a potential transmission route of such symbionts [81]. However, many stinkbugs harbor their symbionts in the crypt cavity of the M4 region instead of the midgut lumen and develop specialized morphological traits of the crypts for their transmission. In the pentatomid stinkbugs, particularly for adult females, several rows of the crypts at the posterior region of the midgut that is morphologically differentiated and conspicuously enlarged, have been found to release the symbiotic bacteria from the crypt cavity to the midgut lumen, thus indicating the insects are able to excrete the symbiotic bacteria from the anus to their egg surface for vertical transmission [82,83]. In acanthosomatid stinkbugs, however, the crypts are completely sealed off, and thus block their connection with the midgut lumen, and the female insect develops a pair of peculiar lubrication organs consisting of numerous bacteria-filled tubules associated with the female ovipositor, therefore facilitating the vertical transmission of the gut symbionts to their eggs by surface contamination [60]. In some subsocial stinkbugs, the female adults have the behavior of tending to their offspring after laying eggs, which enhance the transfer of the gut symbionts during oviposition or near the egg hatching stage. In a subsocial stinkbug of family Cydnidae, the female adults also smear symbiont-containing secretions onto eggs upon oviposition as the non-social stinkbugs [84]. However, in another subsocial stinkbug of family Parastrachiidae, the females start to excrete symbiont-containing materials onto the egg mass before egg hatching, and the newborn nymphs immediately ingest the secretion to acquire the symbionts [85]. Thus, these unique styles of symbiont transfer in turn reduce the risks of microbial inactivation by shortening the exposure duration of the bacteria to the environment.

In addition, some stinkbugs do not directly transfer the symbionts on egg surface and instead have exhibited two unique transmission routes: capsule transmission and jelly transmission (Figure 2B). The plataspid stinkbugs deposit sorts of small brownish particles containing symbionts under the egg mass, so-called “symbiont capsules”, and then newly hatched nymphs feed on the capsules to ensure the inoculation of gut symbiont *Ishikawaella* [86,87]. The insect reinforces the fidelity of this symbiont transmission via the allocation of the capsule number to egg masses at a ratio of about one symbiont capsule per 3.6 eggs, and the capsules that contain a sufficient amount of symbiont cells, about 1.2 ×10^8^ symbionts per capsule [88]. The stinkbugs of the family Urostylididae take another strategy for symbiont transmission, and female adults lay eggs covered with voluminous gut symbiont *Tachikawaea* mixed with supplemented jelly, followed-by newborn nymphs feed solely on the jelly in midwinter and establish the symbiosis. In these species, their basal regions of ovarioles have evolved into specialized structures producing voluminous translucent liquid embedding the mature oocytes. Meanwhile, a pair of symbiont-filled and female-specific organs consisting of glomerate white tubes are associated with the genital chamber. Thus, the symbionts are mixed in the egg covering gelatinous structure during the oviposition to ensure their survival outside the host body several months during the nymphal growth [61]. Collectively, these diverse strategies of symbionts transfer in stinkbugs facilitate reliable vertical transmission over evolutionary time.

### 3.2. The Horizontal Transmission of the Gut Microbiota in the Stinkbugs

Although vertical transmission appears to be predominant among insect–microbe symbioses, horizontal transmission occurs in some stinkbugs. The *Burkholderia* symbionts are widespread in substantial lineages within the superfamilies Lygaeoidea, Coreoidea, and Pyrrhocoroidea, and horizontally acquired de novo from the environment by each host generation [66,79,89]. The *Burkholderia* is specifically localized in the midgut crypts of the stinkbugs and does not transmit to eggs. These insects are aposymbiotic in egg and first nymph stages because of the lack of vertical transmission and orally acquire *Burkholderia* at second instar nymph to establish host–symbiont combinations [89]. Intriguingly, the midgut crypts are rudimentary in the first instar nymph and well-developed in the second instar after oral acquiring the symbiont from the environment, mostly the soils (Figure 2C) [89]. In some largid stinkbugs contain *Burkholderia* species that is phylogenetically close to the plant-associated group rather than the soil group, and the symbiosis is maintained through the environmental acquisition of the bacteria from plant (Figure 2C) [73]. The selectively inoculations of environmental bacteria are determined both by the specialized structure of the host intestine and the microbial traits [90,91]. The stinkbugs develop a specific organ called the “constricted region”, a narrow channel connecting the inner cavities of the M3 and M4 sections of the midgut, which form a favorable passage for the symbiont and blockage other bacteria [11,91]. Meanwhile, the specific lipopolysaccharide O-antigen of the symbiontic *Burkholderia* plays an important role in initialing the symbiotic associations with the host [92], and then the flagella-mediated motility facilitates the migration of *Burkholderia* through the narrow passage of constricted gut region towards the symbiotic crypt region [93]. Soon afterward, the established symbionts trigger the close of the passage and block potential subsequent infection events [94]. In addition, the symbiotic *Burkholderia* accumulate granules of polyhydroxyalkanoate within their cells to confer resistance to nutritional depletion and other environmental stresses for their adaptation to symbiotic conditions [95]. These mechanisms for selective accommodation, and maintenance of their specific microbial partners make the *Burkholderia* horizontal transmission remarkably efficient in *Riptortus pedestris*, in which few bacterial cells per gram of soil are sufficient for the successful inoculation [96].

The combination of vertical and horizontal transmission also has been found in some stinkbugs. In the Blissidae–*Burkholderia* symbiosis, the horizontal acquiring is dominated, whereas up to 30% of the hatchlings vertically acquire symbiotic *Burkholderia* via egg surfaces [97]. The mixed transmission modes are also found in the symbiosis of pentatomoid stinkbug *Plautia stali* and the gut symbiont *Pantoea* spp. The symbionts are primarily vertically transmitted to the egg surface, but some aseptic nymphs also can occasionally acquire suitable free-living *Pantoea* from soil to replace their obligate roles [98].

## 4. Biological Function Roles of Microbial Symbionts in Stinkbugs

Most stinkbugs are phytophagous insects that have a wide array of symbiotic microorganisms that play important roles in nutritional provision and affect the ecologic fitness and even behavior of their insect hosts in many aspects.

### 4.1. The Nutritional Roles of Gut Symbionts in the Stinkbugs

Various stinkbugs are specifically associated with obligate symbionts that are essential for the plant-sap feeding insects. Some members of lygaeoid bugs of the families Blissidae are associated with the bacteriocyte symbionts, such as *K. schneideri* and *S. nysicola,* which are resembled to *Buchnera*-aphids symbiosis, with intracellular habitat and co-evolved with host, have the potentials for supplementing nutrition and benefit their host’s ecological fitness [63]. In addition, most stinkbugs harbor a variety of gut-associated obligate symbionts that play nutritional roles for insect hosts. In plataspid stinkbugs, the gut symbiont *Ishikawaella* generally exhibits drastic genome reduction but retains genes that are capable of synthesizing almost all essential amino acids, some vitamins, and other cofactors that are deficient in the plant sap diet. Experimental removal of the symbionts results in slower growth, higher mortality, and sterility of the insects indicating the essential requirement for their hosts [57,99,100]. Notably, in an urostylidid stinkbug, the jelly transmitted gut symbiont *Tachikawaea* provides their host with essential nutrition to benefit offspring’s growth and survival [61]. In this symbiosis, the females lay eggs covered with voluminous jelly containing symbiont for vertical transmission. The jelly contains both essential and nonessential amino acids proving the sole food source for newborn nymphs to support nymphal growth and survival until the third instar in the winter season, and then the insects establish the obligate associations with *Tachikawaea* to supply essential amino acids when they start to feed on plant sap in spring [61]. Similarly, an obligate gut symbiont *Rosenkranzia* can provide nutrients for fulfilling the development of the acanthosomatid stinkbugs [58].

Pentatomid stinkbugs are generally and obligatorily associated with various bacteria in the genus *Pantoea* in the crypts of the posterior midgut. Among them, *Pantoea carbekii* has the potential to provide supplement essential amino acids and vitamins to its herbivorous hosts *Halyomorpha halys*, thus facilitating their adaptation for a wide range of host plants [101]. However, the effects of the *Pantoea* species on host biology have strong variations with the different associations with pentatomid stinkbugs. The gut symbiotic bacteria are essential for growth and survival in the green stinkbug *Acrosternum hilare*, however, the same symbiont has few fitness on the host and is not necessary for development and survival of *Murgantia histrionica* [102]. The biological roles of the gut symbiont may be different among geographic populations even with the same species. Prado et al. (2006) showed that experimental elimination of the symbiont by sterilizing egg surface and caused few fitness defects in the stinkbug *Nezara viridula* from the Hawaiian population. The symbiont-free insects have normal growth and reproduction similar to the symbiont-present insects in two experiential generations [83]. Whereas, experimental sterilization of the symbiont resulted in severe nymphal mortality for the Japanese population, indicating an obligate host—symbiont relationship [103]. In another pentatomid stinkbug *Plautia stali*, different original populations harbor the obligate symbionts *Pantoea* spp. of several distinct bacterial lineages, including both the uncultivable and cultivable lineages. These symbionts are indispensable for normal host growth and survival, and the sterile newborns can acquire the cultivable symbionts from environmental soils to restore normal growth [98]. Collectively, the *Pantoea* lineages are likely to be an ongoing evolutionary transition from a free-living lifestyle to facultative and obligate mutualism in the pentatomid stinkbugs. The family of Scutelleridae has obligate associations with the gut symbiont *Pantoea* sp. that provides essential roles for insect host survival [76,77].

In addition, the parastrachiid stinkbug *Parastrachia japonensis* has a prolonged (10 months to 2 years) non-feeding diapause at the adult stage, the presence of gut *Benitsuchiphilus tojoi* involve in the supplement of amino acids and vitamins and also have a key role in the recycling of uric acid to aid the host survival the long-term diapause [59,60]. In addition, the symbiont also contains a plasmid encoding genes for thiamine and carotenoid synthesis pathways, which may give the symbiont additional roles in protecting the host against oxidative stress and DNA damage [104].

Some facultative symbionts also have nutritional benefits for their hosts, establishing the important microbes with stinkbugs. In some pyrrhocorid stinkbugs, M3 region of midgut with diverse microbiota includes actinobacterial symbionts (e.g., *Coriobacterium* and *Gordonibacter* sp.) or Firmicutes (e.g., *Clostridium* sp. and *Lactococcus* sp.), and these symbionts essentially affect the survival and growth of their hosts [71,105]. Further study indicates the gut actinobacterial symbionts could also provide B vitamins to their hosts that are scarce in the diet and enhance hosts’ fitness [7]. The vitamin-supplementing symbionts also facilitate their hosts’ adaptation on the plants, and the acquisition of core microbiota enable these insects to be generalist and expand food source to the nutritionally imbalanced and chemically well-defended seeds of Malvales plants [106]. Although the common stinkbug symbiont *Burkholderia* is not strictly obligate and essential for the hosts, several studies show that removal of the symbiont could lead to slower development, lower survival rate, and reduced body weight and size of insect hosts. Moreover, transcriptomic data show the symbionts produced all essential amino acids and B vitamins that are scarce in the host food indicating a nutritional benefit provided by these symbionts [66,79,107].

### 4.2. The Other Ecological Roles of Symbionts in Stinkbugs

In addition to nutritional roles, the gut symbionts may influence the other ecological traits on their hosts, which provide insights into their roles in ecology and adaptation of the insects. The *Burkholderia* are widely found to been capable of insecticide-degradation in the environments and confer the bean bug *R. pedestris* insecticide resistance [13]. The applications of insecticide fenitrothion to agricultural field soils drastically enrich fenitrothion-degrading *Burkholderia*, and the stinkbugs acquire the bacteria from soil during larval development and harbor them in a posterior region of the midgut, resulted in the host with the strong capacity of insecticide resistance [108,109]. The acquisition of *Burkholderia* also effects various aspects of host physiology, including juvenile hormone and the innate immunity, enhancing the reproduction and growth of the insect hosts [110,111,112]. On one hand, the symbiotic *Burkholderia* has a drastic change of bacterial cell envelope that suppresses the immune responses of the symbiotic midgut to prosper the bacteria in their hosts [112]. On the other hand, the inoculation of *Burkhoderia* positively affects the host’s systematic immunity by increasing hemolymph antimicrobial activity and antimicrobial peptide expression in the symbiont-present insects to confer the host better survival [113]. Further, the colonization of *Burkholderia* gut symbiont in the insect also modulate the titer of specific juvenile hormone by increasing the hexamerin-α and vitellogenin proteins in the hemolymph, thus enhancing the reproduction and growth of the hosts [110,114].

Some gut symbionts promote the host behavioral alterations in stinkbugs. In a plataspid bug *Megacopta punctatissima*, newborn nymphs often aggregate and are quiescent after acquiring the gut symbiont *Ishikawaella* from the capsule, whereas these insects keep wandering once the failure of inoculating the symbiont [115]. Genomic analysis also reveals that the symbionts containing a gene encoded for arginine metabolism and oxalate detoxification, and the oxalate-catalyzing enzymes may potentially detoxify plant oxalate to provide a highly adaptive benefits for their insect host [99,116]. Furthermore, the symbionts have been found correlated with the pest status of the insect hosts. Two phylogenetically closely related plataspid stinkbugs, the pest *M. punctatissima* and the non-pest species *M. cribraria*, their pest status on crop legumes are determined by obligate gut symbiont *Ishikawaella* rather than the insect genotype. The exchange of their obligate gut symbionts leads to the reverse of their performance on the crop and pest status [117].

## 5. The Stinkbugs–Gut Microbiota Associations as Targets and Models for Agricultural Pest Control Applications

The insect microbiota has been exploited as targets for pest control, either as delivery agents of insecticide, or use to suppress insect vector competence [118,119]. The major innovation in insect microbiota as the target is to replace the current broad-spectrum chemical insecticides. A recent study of *Wolbachia* inoculations in rice pest brown planthopper has been showed high levels of cytoplasmic incompatibility by reducing the uninfected pest populations and inhibited the transmission of “Rice ragged stunt virus” [120]. In addition, engineering the bacterial symbionts represents another breakthrough in pest control, which has been demonstrated to efficient in both western corn rootworms [121] and honeybee parasitic mites [122]. However, the vast majority of gut microbial symbionts remains unexplored, indicating that the vast majority of the targets also remain untouched—all with promising potential for pest control applications.

Agricultural pests that damage a variety of crop plants globally, particular the stinkbugs cause severe economic impacts on cotton and bean field. The beneficial gut microbial symbionts to enhance the host fitness is not a unique phenomenon of stinkbugs [123,124] but appears to be shared across most agricultural insects. The studies of stinkbug gut microbiota will greatly expand the current well-studied insect–gut microbiota models including *Drosophila*, honeybee, and termite [125,126,127]. In many aspects, the stinkbugs–gut microbiota is valuable to investigate the biological and molecular interactions between host and microbes. In the pyrrhocorid, pentatomid, and plataspid bugs, their gut symbionts are transferred outside during the vertical transmission, and the aposymbiotic and re-inoculation experiments are easily established to analyze their various physiological aspects of hosts, including growth, behavior, metabolism, etc. [7,17,62,71,87]. On the other hand, the obligate partners are essential or important for host survival, development or fecundity, indicating the symbionts may serve as targets for pest control and therefore the disruption of these microbial associations would lead to low vitality of the pest [128]. Specifically, the change of the obligate gut symbionts for a plataspid stinkbug will reverse the pest *M. punctatissima* to be a non-pest status on the legumes crop, indicating the potential microbial targets for pest control [117]. In addition, sorts of gut bacteria have transit stages from free-living to symbiotic, culturable under laboratory conditions, and genetically manipulatable [129,130]. The experimental and genetic tractability of *Burkholderia* in the bean bug *R. pedestris* is a valuable model for engineering the bacterial symbiont as delivery agents of insecticide, similar to the engineered bacterial symbionts in western corn rootworm [121] and honeybee [122], to explore the novel strategy for pest management. Collectively, these features symbiosis in stinkbugs provide the potential models for agricultural pest biocontrol.

## Data Availability

Not applicable.
